# Optimal Dosage of Methylprednisolone for the Treatment of Sudden Hearing Loss in Geriatric Patients: A Propensity Score-Matched Analysis

**DOI:** 10.1371/journal.pone.0111479

**Published:** 2014-11-10

**Authors:** Myoung Su Choi, Ho Yun Lee, Chin Saeng Cho

**Affiliations:** Department of Otorhinolaryngology, Department of Otorhinolaryngology, Head & Neck Surgery, School of Medicine, Eulji University Medical Center, Eulji University, Daejeon, Korea; Sun Yat-sen University, China

## Abstract

We aimed to compare the treatment outcomes and the occurrence rates of adverse events associated with different steroid regimens in geriatric patients (aged 65 years or older) with unilateral idiopathic sudden sensorineural hearing loss (ISSNHL). After thorough medical chart reviews of 109 patients with ISSNHL between May 2006 and December 2013, we performed a propensity score-matched analysis using previously known prognostic factors, steroid regimens, and other cointerventions. Patients were divided based on their steroid regimens into group I (which initially received 48 mg of methylprednisolone daily with a subsequently tapered dose) and group II (which initially received 24 mg of methylprednisolone daily with a subsequently tapered dose). We compared final hearing and the occurrence of adverse events between the two groups. As a result, 20 pairs of propensity score-matched patients (n = 40) were enrolled. Group I patients showed better final hearing levels compared with group II patients (42.00±22.35 dB and 57.38±26.40 dB, respectively), although this difference was marginally significant (p = 0.058). Based on the comparative analysis of each of the frequencies in the final audiograms, lower hearing thresholds at 2 KHz were observed in group I (p = 0.049). There was no significant difference in the occurrence of adverse effects between the two groups (p>0.05). In conclusion, conventional steroid regimens produced adverse event occurrence rates that were similar to those of low-dose treatment but may also have produced superior hearing recovery. The use of steroid dose reduction in geriatric patients with ISSNHL is not preferable to conventional steroid regimens.

## Introduction

According to recent guidelines, initial corticosteroid treatment can be administered to patients with idiopathic sudden sensorineural hearing loss (ISSNHL) [1]. Such treatment is commonly used on the basis of the hypothesis that it may affect the inner ear and induce suppression of the immune response, changes in microcirculation, and a decrease in endolymphatic pressure [2]. However, the effects of steroids on the treatment of sudden hearing loss remain unclear [3]. Although the adverse effects that occur after a 10- to 14-day course of steroids are usually acceptable and manageable [1], various symptoms may occur, including weight gain, gastritis, hypertension, hyperglycemia, cataracts, avascular necrosis of the hip, as well as changes in appetite, mood, sleep patterns, and even death [1], [4], [5].

A study of 18226 patients with diabetes revealed that patients with diabetes and chronic obstructive pulmonary disease who used high-dose corticosteroids were at a greater risk of diabetes-related hospitalization and suggested that the minimally effective corticosteroid dose should be used [6]. More than two-thirds of the geriatric population (aged 65 years or older) have hypertension and 22% to 33% have diabetes, which are associated with a high risk of major complications such as lower-extremity amputation, myocardial infarction, and visual impairment [7], [8]. Therefore, the burden of complications associated with corticosteroid use may be larger in the elderly population than in younger patients.

Prednisone at a dose of 60 mg daily or methylprednisolone (MPD) at a dose of 48 mg for 7 to 14 days is frequently used as an initial medication in the treatment of ISSNHL, and the doses are subsequently tapered [1]. However, many clinicians use slightly different protocols in terms of the type of steroid, dosage, and duration in different clinical settings, and the number of comparative studies of different steroid protocols is limited [1], [9].

Based on these findings, we assumed that, if treatment outcomes following low-dose steroid treatments were as effective as those following higher-dose steroid treatments, the low-dose treatments would be accompanied by a reduced risk of adverse effects and that the use of low-dose steroid treatments for ISSNHL in geriatric patients would be more rational.

In this study, we aimed to compare the treatment outcomes and the occurrence rates of adverse events according to different steroid regimens in ISSNHL patients aged 65 years or older by using a propensity score-matched (PSM) analysis.

## Materials and Methods

### Ethics Statement

This study was approved by the Institutional Review Board of the Eulji University (No. 2014-02-010). The Board granted a waiver of written informed consent for this retrospective study.

### Patients

Based on retrospective medical chart reviews, we enrolled patients who were aged 65 years or older and had been diagnosed with ISSNHL and admitted to the university hospital between May 2006 and December 2013. The exclusion criteria were as follows: 1) concomitant meningitis, myelitis, vasculopathy, or neuropsychiatric disease; 2) a clinical observation period less than 3 months; 3) previous histories of sudden hearing loss and/or the possibility of Meniere's disease; and 4) uncontrolled hypertension or uncontrolled diabetes mellitus.

Age, sex, comorbid diabetes and hypertension, the presence of dizziness and/or tinnitus, the period of time from onset to treatment, the initial hearing levels of both ears, and the final hearing level of the affected side 3 months after the onset of treatment were documented.

### Treatment Protocols

All patients were hospitalized for 1 week, and one of two types of treatment was provided using the time-variant differential approach: either oral MPD treatment or “low-dose” oral MPD treatment. Oral MPD treatment was administered between 2006 and 2008. Subsequently, oral “low-dose” MPD treatment became the mainstream treatment and was commonly used until 2010. From early 2011, an additional intratympanic dexamethasone injection (IT-DEX) administered as a salvage therapy following oral MPD treatment has been the main treatment for sudden deafness.

Patients who received the oral MPD treatment were treated with steroids for 10 days using the same recommended dosage protocol for MPD (48 mg/d for 4 days, followed by a taper of 8 mg every 2 days) [9]. The total cumulative dose of MPD was 432 mg (equivalent to 530 mg of prednisolone) over 14 days. These patients were classified as group I. Patients who received the oral “low-dose” MPD treatment were treated with a half-dose of oral MPD (24 mg/d for the first 4 days, followed by a taper by 8 mg every 2 days). The total cumulative dose of MPD was 144 mg (equivalent to 180 mg of prednisolone) over 8 days. These patients were classified as group II.

Moreover, for patients who required an additional cointervention, a continuous infusion of 10 µg/d of alprostadil over 7 days or daily intravenous infusions of 88 mg of zinc sulfate hydrate were provided.

### Calculation of Hearing Levels and Estimation of Recoveries

Hearing levels were calculated using the arithmetic mean of the hearing levels at 500 Hz, 1 kHz, 2 kHz, and 4 kHz. Hearing improvement rates were calculated as the hearing gain divided by the initial hearing difference between the lesion side and the healthy side and then multiplied by 100 [10], [11]. Complete recovery was defined by a final hearing level within 20 dB or equal to the hearing level of the unaffected ear [10], [11]. Good recovery was defined as hearing gains greater than 30 dB [10], [11]. Fair recovery was defined as hearing gains of 10 to 29 dB. Hearing gains of less than 10 dB were defined as no change or deterioration [10], [11].

### Adverse Events

From the medical chart reviews, insomnia, abdominal discomfort, high blood pressure that occurred more than twice per day (systolic pressure, ≥150 mmHg, and/or diastolic pressure, ≥90 mmHg), and hyperglycemia for which insulin had been newly prescribed or increased were documented. Additionally, major complications such as myocardial infarction, gastrointestinal bleeding, and death were also documented.

### Statistical Analyses

The details of the estimation of the propensity scores were as follows: (1) age, sex, accompanying hypertension and diabetes, presence of tinnitus or dizziness, initial hearing levels of the lesion and healthy sides, IT-DEX treatment, and other cointerventions (alprostadil or zinc injection) were selected as covariates based on the results of the previous studies [12]–[16]; (2) treatment assignment (group I or II) was used as the outcome variable; (3) logistic regression was performed, and propensity scores were calculated. The 1∶1 nearest-neighbor method was used for matching. Next, a test of the balance of the covariates was performed, and the treatment effects were finally compared using paired t-tests and McNemar's tests. All statistical analyses were performed using the SPSS software (ver. 18.0, SPSS Inc., Chicago, IL, USA), and the level of statistical significance was set at a p-value of less than 0.05.

## Results

A total of 109 patients were enrolled in this study, including 44 men (40.4%) and 65 women (59.6%), with a mean age of 71.72 years (range, 65–87 years) and mean ISSNHL duration (from the onset to treatment) of 3.94±2.93 days. Diabetes was reported in 26 patients (23.9%) and hypertension in 56 (51.4%). Of accompanying symptoms, 76 patients (69.7%) had tinnitus and 16 (14.7%) had dizziness. The mean initial hearing level was 68.95±23.26 dB and the initial contralateral hearing level was 43.39±25.00 dB. The baseline characteristics revealed marginally significant differences in age (p = 0.045), accompanying hypertension (p = 0.054), and initial hearing levels (p = 0.071) between the steroid regimen groups.

**Figure 1:**
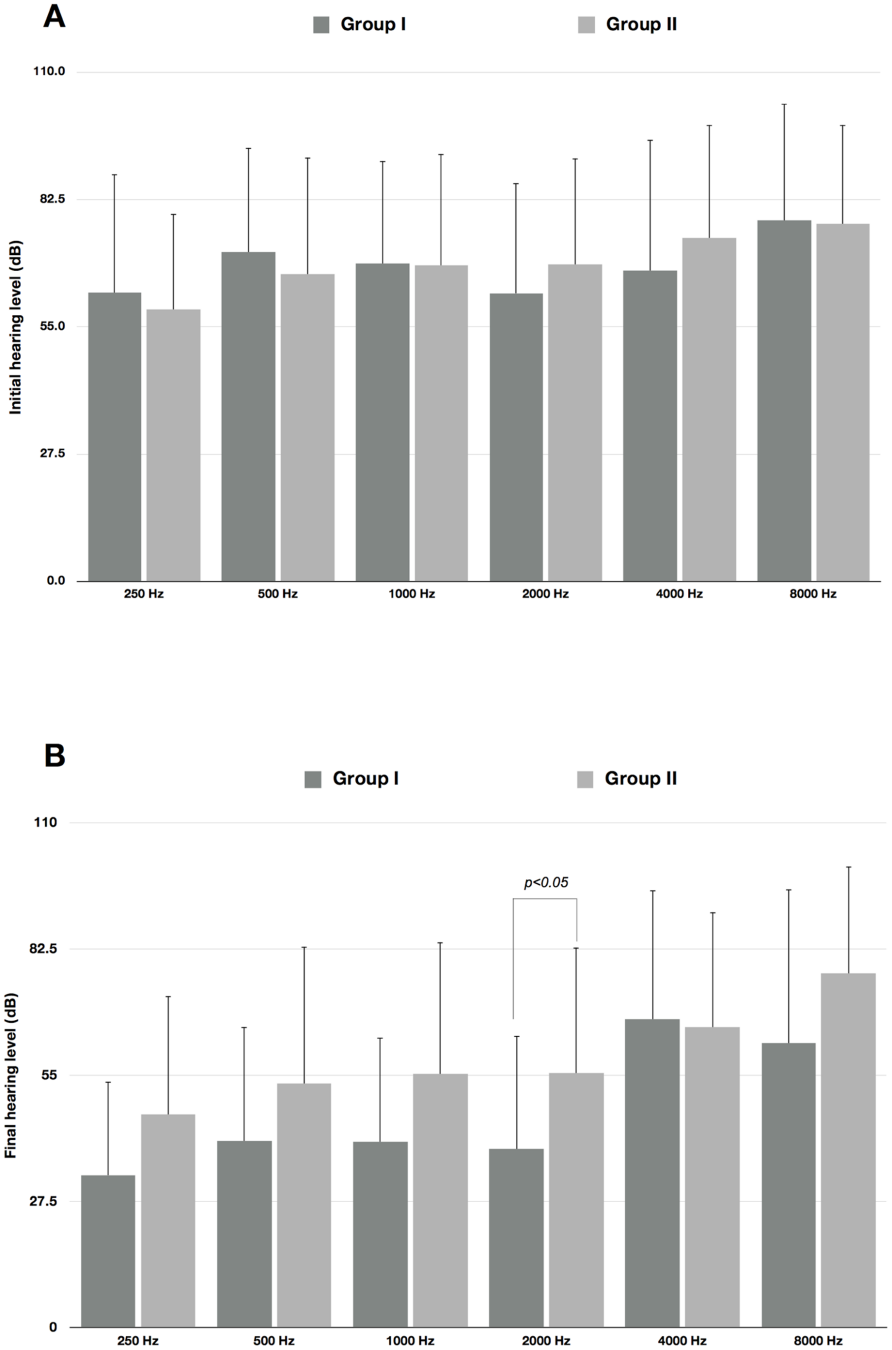
Changes in hearing levels at each frequency before and after treatment in propensity-score matched population. (A) Comparison of the pretreatment hearing levels at each frequency. (B) Comparison of the posttreatment hearing levels at each frequency.

After the PSM analysis, 20 pairs of patients were allocated either to group I or II (Table 1), and the balance test revealed that there were no significant differences in any of the covariates (p>0.05). The final hearing level in group I was 42.00±22.35 dB and that in group II was 57.38±26.40 dB (p = 0.058, 95% confidence interval of the difference: −31.30 dB to 0.55 dB). Comparison of the individual frequencies in the PSM population revealed that the pretreatment audiograms were not different at any of the frequencies between the two groups (Figure 1A, p>0.05); however, the posttreatment audiograms showed marginally significant differences at 1000 Hz (p = 0.065) and 2000 Hz (p = 0.049) (Figure 1B, Table 2).

**Table 1:** Baseline clinical characteristics.

	Total population			Propensity-matched population		
	Group I (n = 66)	Group II (n = 43)	p-value	Group I (n = 20)	Group II (n = 20)	p-value
Age	72.52±5.00	70.49±5.24	0.045	71.45±5.56	71.10±5.15	0.816
Male	26 (59.1)	18 (58.1)	0.798	9 (45.0)	9 (45.0)	1.000
Body mass index (kg/m^2^)	25.10±3.58	24.46±3.14	0.342	25.43±3.87	24.79±3.32	0.555
Diabetes mellitus	17 (25.8)	9 (20.9)	0.563	4 (20.0)	4 (20.0)	1.000
Hypertension	29 (43.9)	27 (62.8)	0.054	10 (50.0)	7 (35.0)	0.337
Days from onset to treatment	3.98±2.73	3.86±3.24	0.829	4.00±3.04	3.40±1.96	0.333
Right side	33 (50.0)	23 (53.5)	0.722	9 (45.0)	9 (45.0)	1.000
Dizziness	12 (18.2)	4 (9.3)	0.200	3 (15.0)	2 (10.0)	0.633
Tinnitus	43 (65.2)	33 (76.7)	0.198	16 (80.0)	15 (75.0)	0.705
Intratympanic injection	5 (7.6)	4 (9.3)	0.749	0 (0.0)	0 (0.0)	–
Alprostadil injection	48 (72.7)	30 (69.8)	0.738	15 (75.0)	18 (90.0)	0.212
Zinc injection	7 (10.6)	1 (2.3)	0.144	0 (0.0)	0 (0.0)	–
Initial hearing (dB)	65.70±23.34	73.92±22.51	0.071	67.38±22.16	69.38±22.25	0.800
Initial contralateral hearing (dB)	43.22±26.89	43.66±22.08	0.928	41.38±27.55	42.63±22.19	0.890
Final hearing (dB)	46.67±26.20	56.54±26.98	0.060	42.00±22.35	57.38±26.40	0.058

Data are presented as mean±standard deviation or number (percentage).

**Table 2:** Comparison of the posttreatment hearing levels at each frequency.

	250 Hz	500 Hz	1000 Hz	2000 Hz	4000 Hz	8000 Hz
Group I (dB)	33.25±20.28	40.75±24.83	40.50±22.65	39.00±24.53	67.25±28.03	62.00±33.50
Group II (dB)	46.50±25.76	53.25±29.84	55.25±28.72	55.50±27.38	65.50±24.92	77.25±23.25
p-value	0.096	0.142	0.065	0.049*	0.864	0.109

Data are presented as mean±standard deviation; *: p<0.05.

The hearing improvement rates did not differ between groups I and II (114.69±299.56% and 123.32±392.28%, respectively; p = 0.941). Regarding hearing recovery, group I showed a tendency for better recovery compared with group II, but the difference was not significant (Table 3, p>0.05).

**Table 3:** Treatment outcomes according to different steroid regimens.

Total population (n = 109)	Total population			Propensity-matched population		
	Group I (n = 66)	Group II (n = 43)	p-value	Group I (n = 20)	Group II (n = 20)	p-value
Recovery, n (%)						
CR	7 (10.6)	1 (2.3)	0.144	2 (10.0)	1 (5.0)	0.370
CR+GR	25 (37.9)	11 (25.6)	0.182	8 (40.0)	3 (15.0)	0.109
CR+GR+FR	42 (63.6)	23 (53.5)	0.291	15 (75.0)	10 (50.0)	0.227
HIR	40.46±270.90	80.13±269.90	0.456	114.69±299.56	123.32±392.28	0.941
Adverse effects, n (%)						
Insomnia	5 (7.6)	1(2.3)	0.400	2 (10.0)	1(5.0)	1.000
Abdominal discomfort	14 (21.2)	12 (27.9)	0.493	3(15.0)	6(15.0)	0.375
High BP	17 (25.8)	15 (34.9)	0.309	7 (35.0)	6 (30.0)	1.000
Hyperglycemia	17 (25.8)	6 (14.0)	0.140	7 (35.0)	4 (20.0)	0.453

CR: complete recovery; GR: good recovery; FR: fair recovery; HIR: hearing improvement rate; BP: blood pressure.

Regarding severe adverse events during treatment, one patient in group II experienced pulmonary edema and upper gastrointestinal bleeding. However, other major complications, such as myocardial infarction and death, did not occur in any of the groups.

Regarding minor adverse events, the PSM analysis revealed that patients in group I complained of insomnia (10% of the patients), abdominal discomfort (15%), high blood pressure (35%), and hyperglycemia (35%). However, the occurrence rates of these adverse events were not significantly different between the two groups (Table 3).

## Discussion

Our study showed that patients receiving conventional steroid treatment had slightly better hearing recovery than patients who received reduced steroid doses, although the difference was marginally significant. Similar trends were found when the final audiograms at all frequencies were compared, with the exception of the 4 kHz frequency. No significant differences in the occurrence rates of adverse effects were found between the two groups.

These findings may suggest that steroid dose reduction is not preferable to the conventional steroid regimen in geriatric patients with ISSNHL.

In contrast, a prospective randomized trial reported that therapeutic outcomes did not differ between 7-day prednisolone and 300 mg dexamethasone pulse therapies [17]. The colleagues of those authors also reported newly developed myocardial infarctions in patients following the pulse therapy and urged clinicians to consider the severe risks of steroid treatment [18]. These findings suggest that the potential benefits of high-dose pulse therapy may not exceed the risks of severe complications. In addition, the duration of steroid use as well as the dosage may be important variables, both of which determine the cumulative steroid dose that may affect the hearing outcome or occurrence of adverse events. The relatively short-term duration of treatment in group II (8 days) compared with that in group I (14 days) might have affected the treatment outcome in our study. A recent survey conducted in the United States reported that 32.2% of the physicians preferred a 14-day steroid treatment, 33.2%, 10-day treatment, and 16.1%, 7-day treatment [19]. This may be attributed to the fact that the optimal dosage and duration of steroid treatment, particularly in elderly population with comorbidities, have not been determined.

With aging, liver function, which is primarily responsible for the metabolism of steroids, is mostly maintained, but phase I metabolism catalyzed by cytochrome P450 tends to decrease, and an increase in interindividual variability is distinctive [20]. Moreover, the affinities of the receptor protein for dexamethasone and corticosterone tend to decrease [21]. Therefore, the use of higher steroid doses might be more rational than lower-dose steroid treatment; however, excessively high-dose steroid treatments have failed to show additional benefits [17]. Based on these findings, we suggest that further studies comparing a greater range of steroid regimens for the treatment of ISSNHL should be performed to identify the optimal dose because we still do not have many options other than steroid treatment [9].

Apart from systemic steroid treatment, intratympanic steroid injection is currently recommended after the failure of the initial treatment [1]. Other possible treatment options include hyperbaric oxygen therapy, and a significant improvement in hearing was reported in the acute stage of ISSNHL following this therapy [22]. The mechanism of action in this therapy is now thought to be the control of cochlear ischemia by increasing oxygen partial pressure. However, in most hospitals, this therapy is not available because it requires a specific sealed chamber. Moreover, it is an expensive and time-consuming treatment method. Other possible options include medications such as antiviral agents [23], vasodilators (such as carbogen, alprostadil, naftidrofuryl, and low-molecular-weight dextran) [24], high-dose vitamins [25], [26], and zinc supplementation [27]. However, the effects of these agents have not been sufficiently studied and there is no evidence to support their use.

To reduce the confounding effects between diverse treatment options and the observed baseline characteristics, we performed the PSM analysis [28], [29]. Selection bias was decreased as far as possible by controlling for the diverse prognostic factors and cointerventions that may have influenced the treatment outcomes. As a result, we were able to compare the intervention effects of the examined steroid regimens by the PSM analysis in order to overcome the limitations of the retrospective, observational study design [29].

Our study has several limitations. It seems unnatural to include covariates such as IT-DEX, alprostadil, and zinc, which may act as confounding factors to the outcome, in the PSM analysis. However, the exclusion of all patients who were treated with cointerventions could result in insignificant conclusions owing to a small sample size. Therefore, we controlled cointerventions as covariates to be able to evaluate the sole effect of steroid dose by minimizing the between-group difference.

In conclusion, conventional steroid regimens produced the occurrence rates of adverse events that were similar to those of low-dose treatment but may also have produced better recoveries. The use of steroid dose reduction in geriatric patients with ISSNHL is not preferable to conventional steroid regimen.
